# Green HPLC strategy for quantification of carvedilol and hydrochlorothiazide in cardiac medications with in-vitro dissolution kinetics and impurity profiling

**DOI:** 10.1186/s13065-025-01559-2

**Published:** 2025-07-03

**Authors:** Mona Nabil, Dina A. Ahmed, Samah S. Abbas, Hayam M. Lotfy, Hoda M. Marzouk

**Affiliations:** 1 Faculty of Pharmacy, Cairo University, El-Kasr El-Aini Street, Cairo, 11562 Egypt; 2 Pharmaceutical Chemistry Department, Faculty of Pharmacy, Future University in Egypt, Cairo, 11835 Egypt; 3 Pharmaceutical Analytical Chemistry Department, Faculty of Pharmacy, Cairo University, El-Kasr El-Aini Street, Cairo, 11562 Egypt

**Keywords:** Blueness, Carbon footprint, Cardiac pharmaceutical formulations, Carvedilol, Click analytical chemistry, Dissolution testing, Greenness profile, Hydrochlorothiazide, Impurity profiling, Whiteness assessment.

## Abstract

The introduction of new pharmaceutical formulations necessitates the development of a trustworthy analytical approach capable of quantifying active ingredients in various quality control procedures. A fixed dosage combination of carvedilol (CAR) and hydrochlorothiazide (HCT) has been introduced to treat hypertension with potential recommendation for diabetic patients. Ahigh-performance liquid chromatographic methodology, designed to be ecologically sustainable while maintaining high precision and accuracy, was established. This approach can simultaneously determine both drugs in their pure forms, and dosage form, along with separation and quantification of potential hydrochlorothiazide related impurities; salamide (DSA) and chlorothiazide (CT). Successful separation was performed using YMC^®^Triart-Phenyl analytical column via gradient elution employing 0.1% formic acid alongside ethanol at a flow rate of 1.0 mL/min, coupled with photodiode array detection at 254.0 nm. Linearity was obtained across the concentration ranges of 0.1 to 100.0 µg/mL for HCT and CAR and 0.05 to 10.0 µg/mL for DSA and CT. The suggested chromatographic methodology can estimate HCT and CAR in different real samples. Additionally, it facilitates the concurrent monitoring of their dissolution profiles. The studied method’s performance was validated in adherence to the guidelines set by the International Conference on Harmonization (ICH). Moreover, its ecological sustainability as well as applicability profile was further affirmed via diverse greenness, blueness, and whiteness assessment tools and compared among official and other reported procedures. In addition, the recently introduced Carbon Footprint Reduction Index tool has been implemented to assess the suggested method with an emphasis on estimating CO_2_ emissions. In general, the suggested methodology proves to be effective for conducting the quality control examination of raw forms and commercially accessible preparations.

**Supplementary Information:**

The online version contains supplementary material available at 10.1186/s13065-025-01559-2.

## Introduction

The worldwide research is focused on establishing analytical approaches that are characterized by ecological friendliness and have the least negative implications on human health, environment, and safety. The green analytical chemistry concepts (GAC) have been established to demonstrate a genuine commitment to protect human health and the environment from the analytical approaches’ negative implications [1, 2]. Furthermore, the white analytical chemistry concept (WAC) [3] is introduced as an advancement of GAC. Beyond green features, WAC also incorporates another critical factors influencing the method quality, encompassing analytical efficacy (Red), practical (Blue). This posed a challenge for researchers to develop a multi-criteria methodology for evaluating the comprehensive analytical procedure, encompassing safety, performance, and ecological impacts. The newest concepts of blueness were also adopted in accordance with sustainable environmental principles [4]. Serving as a complement to established green metrics, it primarily emphasizes the practical perspectives aligned with the aspects of WAC.

Cardiovascular disease is one of the top causes of illness and death globally. Hypertension poses a significant worldwide health concern, impacting millions of individuals for being the foremost avoidable risk factor for atherosclerosis and ischemic heart disease [5]. Nowadays, hypertensive patients may require a combination of antihypertensive medications instead of utilizing a single antihypertensive treatment to ensure a significant decrease in blood pressure records, improved target organs’ protection, with a concurrent decrease in adverse effects [6]. It is worth knowing that hypertension and type 2 diabetes usually manifest as comorbidities. The occurrence of hypertension is double in individuals diagnosed with diabetes, in contrast to non-diabetic individuals. On the other side, individuals who have hypertension usually demonstrate the symptoms of insulin resistance and have a higher susceptibility to diabetes, in contrast to those without hypertension. Moreover, there is considerable similarity in hypertension and diabetes cardiovascular complications, principally concerning macrovascular and microvascular disease [7, 8].

For decades, the formulations’ dissolution testing has been established as a descriptive test and an essential tool usually employed within the pharmaceutical industry. This emerges due to its capacity to depict the in-vivo drug performance via measuring the in-vitro rate of drug release over time. This in turn will govern authenticating the product’s consistency and assuring dosage forms’ performance [9, 10, 11]. Traditionally, dissolution testing was performed using UV spectrophotometry. However, several obstacles were faced, including: the use of spectrophotometry is limited to active ingredients with chromophore groups, spectral overlap can represent challenges when dealing with mixtures of analytes, limited sensitivity and selectivity, and turbid solutions formed during testing the dissolution may complicate spectrophotometric analysis [12, 13, 14, 15]. As a result of all these problems, high-performance liquid chromatographic (HPLC) methodology excelled in the field of scientific research for being an attractive, powerful, and dependable alternate in biological investigations and in dissolution testing of pharmaceutical preparations. In comparison to the frequently employed conventional spectrophotometric approach, HPLC offers the unique advantages of; high sensitivity and selectivity, time saving, amenity to automation, high versatility in compounds’ analysis, efficiency for large-scale production, and impurity profiling enabling both qualitative and quantitative detection of impurities to assure the final product’s quality, safety and efficacy [16, 17, 18, 19, 20].

**Fig. 1:**
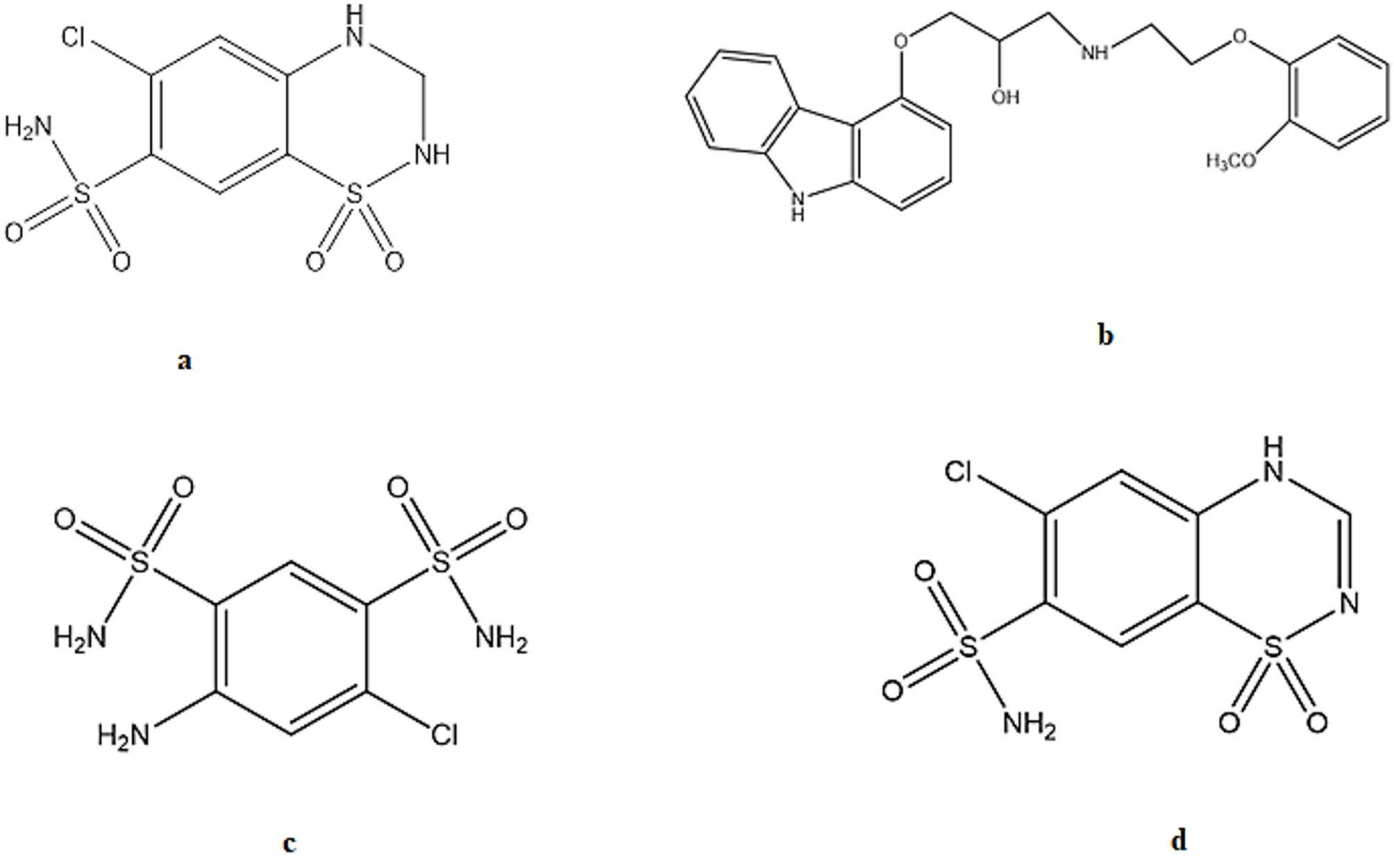
Chemical structures of (**a**) Hydrochlorothiazide (HCT), (**b**) Carvedilol (CAR), (**c**) Salamide (DSA) and (**d**) Chlorothiazide (CT)

Hydrochlorothiazide (HCT); Fig. 1a is categorized as a thiazide diuretic that inhibits the electroneutral Na^+^–Cl^–^ cotransporter situated on the apical surface of the distal convoluted tubule’s initial segment [21]. Carvedilol (CAR), Fig. 1b, is usually prescribed for individuals with diabetes to manage congestive heart failure and hypertension. It exhibits α_1_ and non-selective β antagonistic action alongside its moderate calcium channel blocking capabilities [22]. Compared to a traditional beta-blockers regimen, CAR lowers blood pressure via diminishing vascular resistance whilst sustaining cardiac output and has a milder impact on heart rate. For that reason, CAR is a drug of choice for hypertensive diabetic patients as it improves glucose and lipid metabolic parameters by reducing lipid peroxidation [23, 24, 25]. Co-Dilatrol^®^Tablet is a cardiovascular medication that comprises HCT and CAR. Taken together, a pronounced pharmacological impact is achieved to control the elevated blood pressure in the shortest possible time with maximum patient’s satisfaction [26].

Identification and quantification of impurities is required by different regulatory authorities as it is a pivotal aspect in the development of pharmaceutical processes [16, 17, 18, 19, 20, 27]. The British pharmacopeia considered that Salamide (DSA), 4-amino-6-chlorobenzene-1,3-disulphonamide; Fig. 1c, and chlorothiazide (CT), 6-chloro-2 H-1,2,4-benzothiadiazine-7-sulphonamide-1,1-dioxide; Fig. 1d, to be the main process-derived impurities or products of degradation associated with HCT (HCT impurity B and A, respectively) [28]. DSA was found to have certain carcinogenic activity, while the pharmacological activity of CT was found to be lower than that of the parent drug; HCT [29].

Literature review revealed that both HCT and CAR in their combined tablet dosage form have been estimated via spectrophotometric [30, 31, 32, 33, 34], HPLC [35, 36], HPTLC [37], and CE [38] methods. Up to the authors’ recent knowledge, no previous methodology that is both time-efficient and eco-friendly has been reported for the concurrent analysis of the investigated analytes, along with the impurities related to HCT within a single run, a crucial requirement for pharmacotherapeutic drug monitoring, impurity profiling, and the quality control investigation of pharmaceuticals. Therefore, this research’s principal objective is to establish an efficient, robust, economic, and ecologically sustainable HPLC-DAD methodology for the concurrent quantification and purity assessment of the studied analytes in both their raw forms and pharmaceutical preparations. Furthermore, a successful in-vitro dissolution monitoring of Co-Dilatrol^®^ Tablets was accomplished. Moreover, the method’s ecological sustainability and applicability were comprehensively evaluated by employing various up-to-date greenness, blueness, and whiteness assessment metric tools in parallel to previously reported methods [35, 36].

## Experimental

### Instrumentation

The Waters Alliance HPLC instrumentation comprises an alliance quaternary pump from the model 2690 series was produced by Waters Corporation (Milford, MA, USA), offering variable flow rate, equipped with a Waters 996 photodiode array UV detector, a vacuum degasser, and an auto-sampler coupled with 100- µL loop was employed. Data acquisition, processing, and reporting were performed via utilizing **Empower™** software, which was produced by Waters Corporation (Milford, MA, USA). The Vankel VK 7000 device was produced by Varian, Inc., acquired by Agilent Technologies in the United States, was equipped with six vessels, and a standard USP type-II paddle was used for invitro-dissolution monitoring of Co-Dilatrol^®^ Tablets.

### Materials and reagents

Ethanol of HPLC-grade and formic acid were supplied by Merck (Darmstadt, Germany). Double-distilled deionized water was procured from Sigma Aldrich Chemie (Darmstadt, Germany).

### Samples

Hydrochlorothiazide (HCT) and carvedilol (CAR) pure samples were supplied from Global Napi Pharmaceutical Industries (Cairo, Egypt). Purities were verified to be 99.88% ± 0.52 for HCT and 99.93% ± 0.62 for CAR according to their official methods [28], respectively. HCT’s related impurities; DSA and CT, were procured from Sigma Aldrich Chemie (Darmstadt, Germany) with respective purities of 99.80% and 99.60%. Co-Dilatrol^®^Tablet dosage form (Lot No.070140 A) was manufactured by Chemipharm (Cairo, Egypt). The unit dose encompasses 12.5 mg HCT and 25.0 mg CAR.

### Standard solutions

Stock solutions were individually prepared at a concentration of 1.0 mg/mL for HCT, CAR, DSA, and CT by dissolving 100.0 mg of respective analyte with ethanol as a solvent into 100-mL volumetric flasks.

### Procedure

#### Chromatographic conditions

The chromatographic separation procedure was conducted at room temperature utilizing a YMC^®^ Triart-Phenyl column (150 × 4.6 mm, 5 μm) (YMC America Inc., USA), employing a gradient elution program with a mobile phase comprising solvents A (0.1% formic acid) and B (ethanol). The gradient program (time /%B) was set as 0–5.0/20.0%, then step up gradient of mobile phase B from 5.0 to 7.0 min reached 80.0%, and during the interval of 7.0 to 10.0 min it remained 80.0%. Following a period of 10.0 min, the gradient program was reverted to its original state, and the analytical column underwent reconditioning for a duration of 5 min. The mobile phase underwent filtration utilizing a Millipore membrane filter with a particle size of 0.45 μm and was dispensed at a flow rate of 1.0 mL/min. A volume of 50.0 µL was injected utilizing a 250 µL analytical syringe. The diode array detector was employed for monitoring the eluent at a wavelength of 254.0 nm.

#### Calibration curves’ construction

Portions from 1.0 to 1000.0 µg for both HCT and CAR and from 0.5 to 100.0 µg for both DSA and CT were relocated from their respective stock solutions (1.0 mg/mL) into four sets of 10- mL volumetric flasks. The volumes were made up to the mark utilizing the mobile phase to get final concentrations of 0.1–100.0 µg/mL for both HCT and CAR and 0.05–10.0 µg/ mL for both DSA and CT. For the resulting solutions, the aforementioned chromatographic conditions were employed in triplicate, and the chromatograms were subsequently stored. Calibration curves were built up through the correlation of the corresponding average peak areas with the matching concentrations, followed by the determination of each component’s relevant regression equation.

#### Laboratory prepared mixtures’ assay

The studied method’s selectivity was verified via analyzing laboratory prepared mixtures comprising various proportions of the mentioned drugs in their raw forms. Appropriate portions of pure HCT, CAR, and the HCT impurities; DSA and CT were precisely relocated from their relative stock solutions into sets of 10-mL volumetric flasks, diluting to the calibration mark utilizing the mobile phase. The aforementioned chromatographic conditions were employed. Afterwards, the equivalent regression equations were employed to compute the concentrations.

#### Application to pharmaceutical formulation

Ten Co-Dilatrol^®^ Tablets were weighed individually, crushed, and uniformly blended. A quantity equivalent to the weight of one tablet was precisely placed into a 100-mL beaker, following that 50.0 mL of ethanol was added, and subsequently sonication was conducted for a duration of 30 min. The resultant solution underwent filtration into a 100-mL volumetric flask, and the volume was adjusted to the calibration mark utilizing ethanol. Additional dilution was conducted via relocating 8.0 mL into a 100-mL volumetric flask and then filling it to the calibration mark utilizing the mobile phase. The ultimate concentrations were 10.0 µg/mL for HCT and 20.0 µg/mL for CAR.

#### Dissolution kinetics

The abovementioned HPLC-DAD approach was employed to monitor the in-vitro dissolution profiles of Co-Dilatrol^®^ tablets in 0.1 N HCl following the USP-specification [39]. Dissolution kinetics was conducted by introducing a single tablet into a USP dissolution apparatus II vessel, encompassing a volume of 900 mL of 0.1 N HCl as the dissolution medium. The system was subsequently maintained at 37 ^◦^C ± 0.5^◦^C and run at a speed of 50 rpm. At specified time points of 10.0, 20.0, 30.0, 45.0, and 60.0 min, samples of five milliliters were taken, and the withdrawn volume was replaced with an equivalent volume of fresh medium. Following collection, the samples underwent filtration utilizing a syringe filter with a pore size of 0.22 μm. The test was replicated three times. The percentage dissolution was determined utilizing each component’s relevant regression equation in the investigated medium, followed by the plotting of the in-vitro release profiles for HCT and CAR. Suitable dilutions from stock solutions of HCT and CAR were made individually into two sets of 10-mL volumetric flasks utilizing 0.1 N HCl as a solvent. Calibration curves were subsequently constructed, followed by the determination of each analyte’s relevant regression parameters.

## Results and discussion

Maintaining the safety and quality of the pharmaceuticals in drug manufacturing control necessitates comprehensive chemical and analytical support across all production stages. The HPLC separation technique is the primary approach utilized across the multiple phases of pharmaceutical development and manufacturing. Identification and quantification of impurities is required by different regulatory authorities as it is a pivotal aspect in the development of pharmaceutical processes. Moreover, the safety and effectiveness of the pharmaceutical preparations can be affected by the existence of these undesirable components even in trace quantities. Thus, the principal objective of this research is to establish an efficient, robust, sensitive, and ecologically sustainable methodology for the concurrent quantification and purity assessment of HCT and CAR, along with HCT’s related impurities for the first time.

The suggested analytes’ dissolution profiles in the combined pharmaceutical preparation were also assessed employing the studied HPLC-DAD methodology. Finally, various green, blue, and white assessment tools were exploited for evaluating the proposed method’s ecological, health implications and applicability.

### Challenges in the separation of the investigated analytes

The structure’s similarity between HCT and CT resulted in difficulty in separation between them. Moreover, it is a challenge to achieve a complete separation of all four analytes in the shortest run time less than 9 min.

### Optimization of chromatographic conditions

The impact of various variables on enhancing the optimization of the HPLC-DAD methodology was assessed. Different organic solvents, including acetonitrile, methanol, and ethanol as well as acetate buffer, phosphate buffer, and 0.1% formic acid as aqueous phases, were examined with diverse ratios, flow rates, and pH values. Acetonitrile was excluded to enhance the eco-friendliness of the methodology. While ethanol was chosen because it gave better resolution with a stable baseline, as well as being a green solvent. Moreover, phosphate and acetate buffers (pH; 3.0, 4.0 & 5.0) were also excluded as they gave poor resolution between HCT and CT; HCT’s related impurity with a delayed elution of CAR.

Multiple trials were conducted to select the better mobile phase composition. Numerous isocratic programs utilizing ethanol and methanol, each with 0.1% formic acid in varying ratios, were executed, but poor resolution and broad peaks were observed. Furthermore, multiple gradient programs encompassed 0.1% formic acid as solvent A, and ethanol as solvent B, with diverse ratios were attempted. Upon utilizing a gradient elution system commencing with 20% v/v solvent B from zero to 5.0 min, then ramping up to 80% v/v solvent B from 5.0 to 10.0 min, a satisfactory separation was attained, but a delayed elution of CAR was observed. The optimal balance among appropriate peak shapes, sufficient resolution, and feasible retention times was attained utilizing the same mentioned gradient elution system, but with an early ramping up to 80%, from 5 to 7 min, then keeping the percentage of solvent B constant till 10 min. Then the gradient program was reset to its original state, and the YMC^®^ Triart-Phenyl column underwent reconditioning for a duration of 5 min.

The mobile phase was dispensed at variable flow rates; 1.0, 1.5, and 2.0 mL/min. Whereas, the proper flow rate of 1.0 mL/min was found to offer adequate separation with appropriate analysis time and satisfactory resolution. In the aforementioned isocratic and gradient programs, diverse stationary phases, including YMC^®^ Triart-Phenyl, YMC^®^ C_8_, and YMC^®^ C_18_ columns, were examined. For the YMC^®^ C_8_ column (100 × 4.6 mm, 5 μm), poor resolution was achieved between the studied analytes, as it gave a resolution less than 1.5, which is out of the acceptable limit, meanwhile, broad peaks were observed with a tailing factor more than 2. Upon using the YMC^®^ C_18_ column (150 × 4.6 mm, 5 μm), CAR exhibited high retention on the column, as it has a retention time equal to 14 min, which resulted in prolonged run time; also, a marked peak tailing was attained with a tailing factor more than 2. The highest resolution and most favorable system suitability parameters were attained via utilizing YMC^®^ Triart-Phenyl column (150 × 4.6 mm, 5 μm) with resolution achieved to be more than 1.5 among the investigated analytes, with selectivity value more than 1 (˃ 1.1 for optimum separation), tailing factor less than 2, and column efficiency more than 2000. Additionally, this column was chosen as it is characterized by unique selectivity, excellent durability and stability, and better resolution and peak shape without tailing. The wavelength range selected for HPLC UV-VIS detection plays a key role in determining analytical performance. Factors such as sensitivity, selectivity, linearity, and method stability are affected by this choice. A diode array detector was utilized, and several wavelengths were tried (210.0, 254.0, 242.5, 285.5, 226.1, and 270.5 nm). The most suitable wavelength was 254.0 nm as it exhibited optimum sensitivity and peak symmetry for all components, as well as minimum noise sharpness.

### System suitability

The effectiveness of the chromatographic approach was confirmed through the computation of system suitability parameters as per USP standards set [39], which are crucial for evaluating its effectiveness. Whereas, these parameters were determined and sufficient outcomes were attained with suitable retention times (t_R_), selectivity factors (α) achieved to be 1.68, 1.12, and 2.10 for DSA, HCT, CT, and CAR, respectively, with experimental resolutions (Rs) of 6.02, 1.65, and 19.78 for DSA, HCT, CT, and CAR, respectively, while, tailing factors (T) were determined to be 1.17, 1.07, 1.33, and 1.28 for DSA, HCT, CT, and CAR, respectively, with theoretical plate counts (N) computed to be 4990, 5930, 6990, and 31,800 for DSA, HCT, CT, and CAR, respectively, ultimately heights equivalent to theoretical plate (HETP) were 3.00 × 10^− 3^, 2.53 × 10^− 3^, 2.15 × 10^− 3^, and 4.72 × 10^− 4^ cm/plate for DSA, HCT, CT, and CAR, respectively as depicted in Table 1.

**Table 1:** System suitability parameters of the proposed HPLC-DAD method

Parameter	DSA	HCT	CT	CAR	Reference value [39]
Retention time (t_R_) (min)	3.4 ± 0.03	4.69 ± 0.04	5.06 ± 0.03	8.96 ± 0.02	
Selectivity (α) ^a^	1.68	1.12	2.10		> 1
Resolution (Rs) ^b^	6.02	1.65	19.78	N/A	> 1.5
Tailing factor (T)	1.17	1.07	1.33	1.28	T ≤ 2
Column efficiency (N)	4990	5930	6990	31,800	*N* > 2000
Height equivalent to theoretical plates (HETP) (cm/plate)	3.00 × 10^− 3^	2.53 × 10^− 3^	2.15 × 10^− 3^	4.72 × 10^− 4^	

^a^ Selectivity (α) = *k’*_2_/*k’*_1_ calculated for each of two successive peaks

^b^ Resolution (R_s_) = 2(t_RB_ - t_RA) / (_W_B+_W_A_), and w is the peak width calculated for each of two successive peaks

**Fig. 2:**
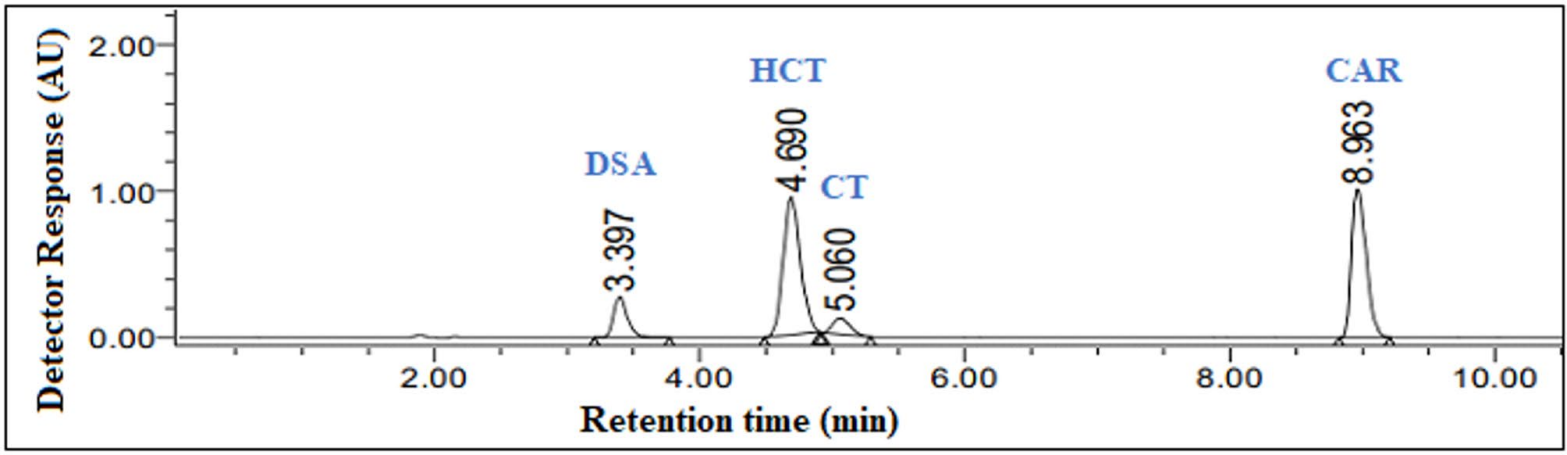
HPLC-DAD chromatogram of a mixture of DSA (5.0 µg/mL), HCT (50.0 µg/mL), CT (5.0 µg/mL) and CAR (50.0 µg/mL) using gradient elution system of 0.1% formic acid and ethanol at 254.0 nm

Ultimately, upon employing these optimal chromatographic conditions, a satisfactory separation and sharp peaks without any tailing were obtained for HCT and CAR, along with the impurities associated with HCT with retention times achieved to be 3.4 ± 0.03 min, 4.69 ± 0.04 min, 5.06 ± 0.03 min and 8.96 ± 0.02 min for DSA, HCT, CT and CAR, respectively. The total analysis time required for achieving complete separation of all four analytes was approximately 10 min; Fig. 2.

The specificity of detection was affirmed with the assistance of a diode array detector, achieved by individually detecting each analyte’s retention time and investigating the diverse components’ UV spectra at various retention times.

### Method validation

The developed HPLC methodology was validated in adherence to the ICH guidelines [40], encompassing sensitivity, linearity range, accuracy, precision, LOD, LOQ, and robustness with minor modifications applied to the flow rate (± 0.1 mL/min) and scanning wavelength (± 1 nm) as depicted in Table 2.

**Table 2:** Regression and validation parameters of the proposed HPLC-DAD method

Method Parameter	DSA	HCT	CT	CAR
**Calibration range (µg/mL)**	0.05-10.0	0.1–100.0	0.05-10.0	0.1–100.0
**Regression equations parameters**				
**Slope (b)** ^a^	339,369	187,578	141,407	135,798
**Intercept (a)**	14,505	137,700	18,724	58,515
**Correlation coefficient (r)**	0.9999	1.0000	1.0000	1.0000
**Accuracy (Mean ± SD)**	99.13 ± 0.54	100.99 ± 0.75	100.39 ± 0.77	100.82 ± 0.42
**Precision**				
**± %RSD** ^b^	0.29	0.46	0.80	0.15
**± %RSD** ^c^	1.07	1.15	1.09	0.91
**LOD (µg/mL)** ^d^	0.01	0.03	0.01	0.02
**LOQ (µg/mL)** ^d^	0.03	0.09	0.03	0.06
**Selectivity (mean ± SD)**	99.53 **±** 0.78	100.55 **±** 0.53	99.92 **±** 0.97	99.55 **±** 0.65
**Robustness** ^e^	0.52	0.15	0.43	0.21

^a^ Regression equation: *A* = *a* + *b c*, where ‘A’ is the average peak area and ‘c’ is the concentration

^b^ Intra-day precision [average of three different concentration of three replicates each (*n* = 9) within the same day], the concentrations were: (20.0, 40.0, 60.0 µg/mL) for HCT and CAR and (2.0, 4.0, 6.0 µg/mL) for DSA & CT

^c^ Inter-day precision [average of three different concentration of three replicates each repeated on three successive days], the concentrations were the same as in intra-day precision

^d^ LOD and LOQ are calculated according to ICH, 3.3 × SD of the residuals/slope and 10 × SD of the residuals/slope, respectively

^e^ Average %RSD of the change in detection wavelength (± 1 nm) and flow rate (± 0.1 mL/min)

Table S1 demonstrates the findings attained from investigating laboratory-prepared mixtures encompassing HCT and CAR, alongside varying ratios of the impurities related to HCT, thereby verifying the mentioned method’s specificity, where favorable outcomes were achieved within the established calibration ranges. Moreover, the suggested methodology was employed for assaying HCT and CAR in their pharmaceutical formulation; Co-Dilatrol^®^ tablets. Additionally, the method’s validity underwent assessment via employing the standard addition technique; Table 3.

**Table 3:** Results obtained by applying the proposed HPLC-DAD method for the determination of HCT and CAR in Co-Dilatrol^®^ tablets and application of standard addition technique

Pharmaceutical formulation		% Found ± SD ^a^	Standard addition technique		
			Claimed (µg/mL)	Pure Added (µg/mL)	% Recovery of the pure added ^b^
**Co-Dilatrol**^®^**Tablets** (B. No. 070140 A; labeled to contain 12.5 mg HCT and 25.0 mg CAR per tablet)	**HCT**	100.10 ± 0.82	10	5.00	99.00
				10.00	100.60
				20.00	100.75
			**Mean ± SD**		**100.12 ± 0.97**
	**CAR**	99.58 ± 0.56	20	10.00	99.20
				20.00	100.85
				40.00	100.68
			**Mean ± SD**		**100.24 ± 0.91**

^a^ Average of five determinations

^b^ Average of three experiments

### Statistical analysis

Upon statistically comparing the findings attained from analyzing HCT and CAR in their raw forms employing the studied HPLC methodology against those findings acquired from employing the official approaches [28], it was observed that the determined *t* and F values were below their equivalent tabulated values at *P* ≤ 0.05. This indicates that, in regard to precision and accuracy, no substantial variation was detected; Table S2. Finally, one-way ANOVA testing was executed to statistically compare the outcomes attained by the suggested methodology with those achieved by the official approaches. This analysis revealed no substantial variation as the F value is below the critical one and the P value > 0.05; Table S3.

### Greenness profile assessment

The assessment of the analytical procedures’ greenness has acquired an increased significance. The principal goal of this assessment is to promote ecological sustainability while ensuring the method’s effectiveness by removing or minimizing harmful chemicals in analytical methodologies [41, 42, 43, 44, 45, 46, 47, 48, 49]. Therefore, the method’s ecological sustainability profile was appraised through up-to-date and reliable metric tools, namely; analytical GREEness (AGREE) [50], modified green analytical procedure index (MoGAPI) [51], and Analytical Green Star Area (AGSA) [52], in comparison to the official methods [28] as well as to previously reported HPLC ones [35, 36].

**Fig. 3:**
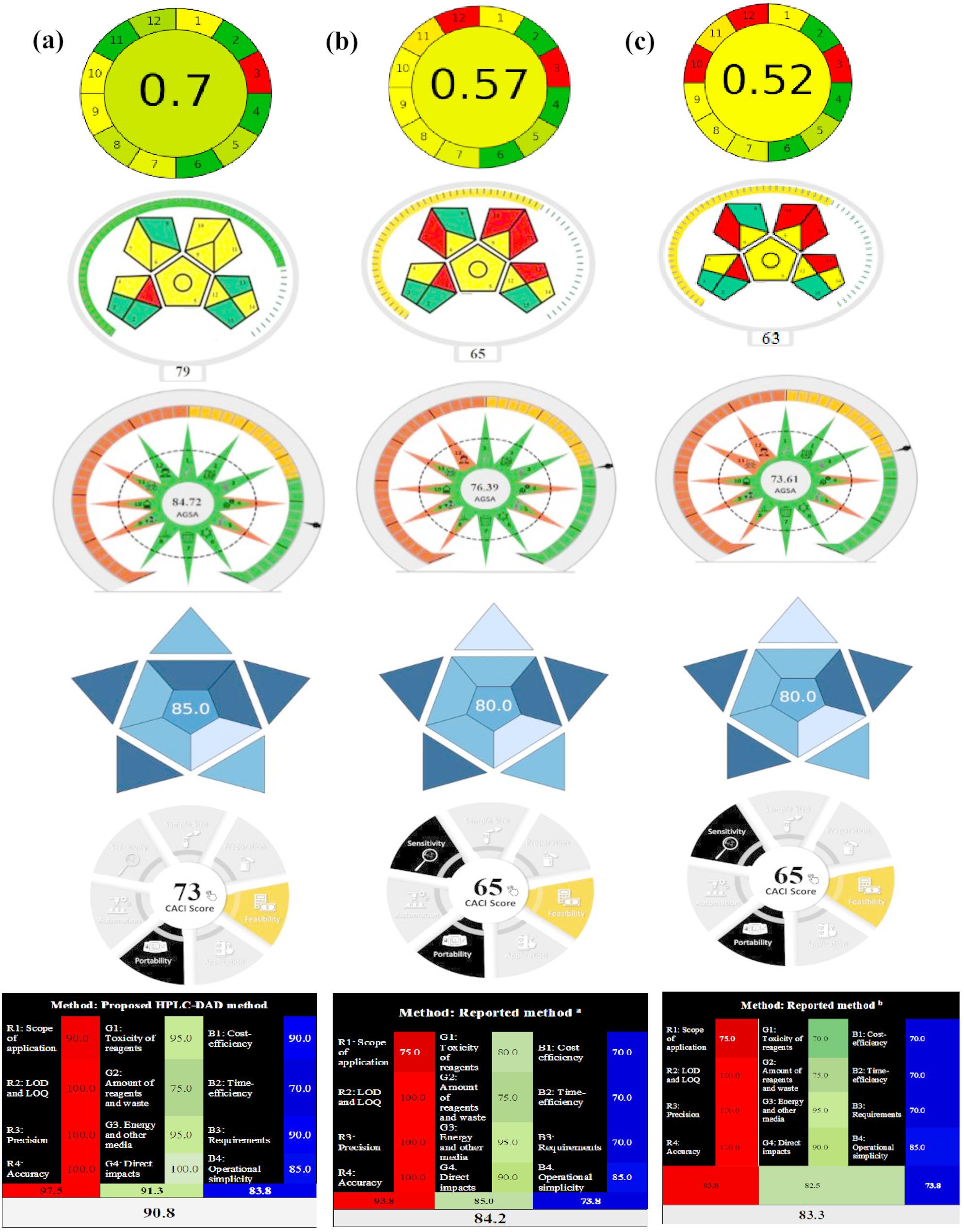
Greenness, blueness and whiteness assessment of (**a**): the proposed HPLC-DAD method, (**b**): Reported HPLC method [35] and (**c**): Reported HPLC method [36], via AGREE, Mo GAPI, AGSA, BAGI, CACI and RGB 12 assessment tools

AGREE is an analytical greenness metric software, which is freely available at https://mostwiedzy.pl/AGREE, and provides a comprehensive assessment of all 12 GAC principles by generating a pictogram that contains the overall score as a fraction of unity, ranging from zero to one. Upon applying this tool, the HPLC-DAD method described in this study achieved a higher AGREE score (0.7) compared to the official methods of HCT (0.57) and CAR (0.53), as well as the two previously reported HPLC ones wherein pictogram’s score values were 0.57 and 0.52, Fig. S1 and Fig. 3.

MoGAPI software, freely available at bit.ly/MoGAPI, offers a more precise assessment of greenness. It not only provides the typical red/green/yellow pictograms of the commonly used GAPI tool but also provides an overall assessment of the method greenness by calculating a total score. As shown in Fig. S1 and Fig. 3, the suggested approach achieved higher score (79) which makes it overall excellent green (≥ 75) compared to the official methods and reported ones.

Finally, the new AGSA tool, freely available at bit.ly/AGSA2025, introduces a comprehensive, built-in scoring and visual representation approach structured around 12 principles of green analytical chemistry. The greater the green area of an analytical method in AGSA, the higher the level of greenness. According to Fig. S1 and Fig. 3, it can be observed that the proposed HPLC-DAD method exhibits a higher level of environmental friendliness when compared to the official and reported methods.

From the above-mentioned assessment tools, the proposed HPLC method proved its preference over the official methods for determining HCT and CAR [28] and the reported approaches [35, 36]; Fig. S1 and Fig. 3, and this is attributed to the consumption of lower instrumental energy and mitigation of solvent usage via utilizing ecologically sustainable solvents as ethanol, as proved by achieving elevated scores for AGREE (0.7), MoGAPI (79), and AGSA (84.72).

### Whiteness tool for evaluating the analytical method

The three core pillars of sustainable development are integrated; analytical efficacy, green features, and practical and economic attributes. The novel RGB12 model [3] serves as a quantitative assessment tool for providing sustainability in the universal analytical approach’s assessment via determining the whiteness scores of the analytical approach [3, 42, 43, 53, 54, 55, 56, 57, 58, 59]. This model utilizes three colors; red, green, and blue colors. Red is designated to represent analytical effectiveness, as determined by validation criteria encompassing sensitivity, accuracy, precision, LOD, and other relevant parameters, green signifies adherence to GAC concepts concerning ecological impacts, encompassing, the quantity and amount of the reagents, the toxicity of utilized reagents, waste yielded throughout the process, energy consumption, and overall ecological impact. Whilst blue signifies economic and practical attributes, encompassing factors such as time, cost, minimal requirements, and operational simplicity. To assess the analytical approach, it is crucial to complete the red, green, and blue tables provided in the Excel spreadsheet [3]. After undergoing computational appraisal, the methodology is allocated a color, determined by the distribution of three key colors. An optimal, efficient, reliable, and sustainable methodology produces a white color via highly saturating all three colors. The model facilitates concurrent appraisal, where a score of 0 represents the least favorable finding, and a score of 100 signifies that the method is well-suited for application concerning a specific concept.

As declared in Fig. S1 and Fig. 3, the WAC tool demonstrated superior analytical performance of the proposed HPLC-DAD method over the official and published approaches due to broader range of applications, shorter analysis time, lower energy consumption, reduced use of hazardous solvents, and use of sustainable bio-based solvents (ethanol) as proved by achieving the highest whiteness score (90.8%).

### Carbon footprint reduction index tool

The CaFRI [60] is a broad greenness assessment tool that emphasizes the carbon footprint as the principal environmental impact, specifically designed for evaluating established analytical laboratory procedures. The assessment considers multiple critical factors, including energy consumption, the carbon dioxide production, implementation of targeted carbon footprint reduction strategies, sample storage, transportation, personnel involvement, waste management, recycling initiatives to reduce resource consumption, and chemical usage. The highest score achievable by any analytical method within the CaFRI assessment is 100. The tool is integrated with the user-friendly software, which is freely accessible online at (https://bit.ly/CaFRI). Various regions on the footprint diagram correspond to specific criteria, with red indicating a poor rating, yellow representing an average rating, and green signifying a favorable rating, consistent with the principles of green chemistry. The points accumulated from the questionnaire are translated into a final score ranging from 0 to 100, where a fully green procedure, in terms of carbon footprint evaluation, achieves the maximum score of 100.

**Fig. 4:**
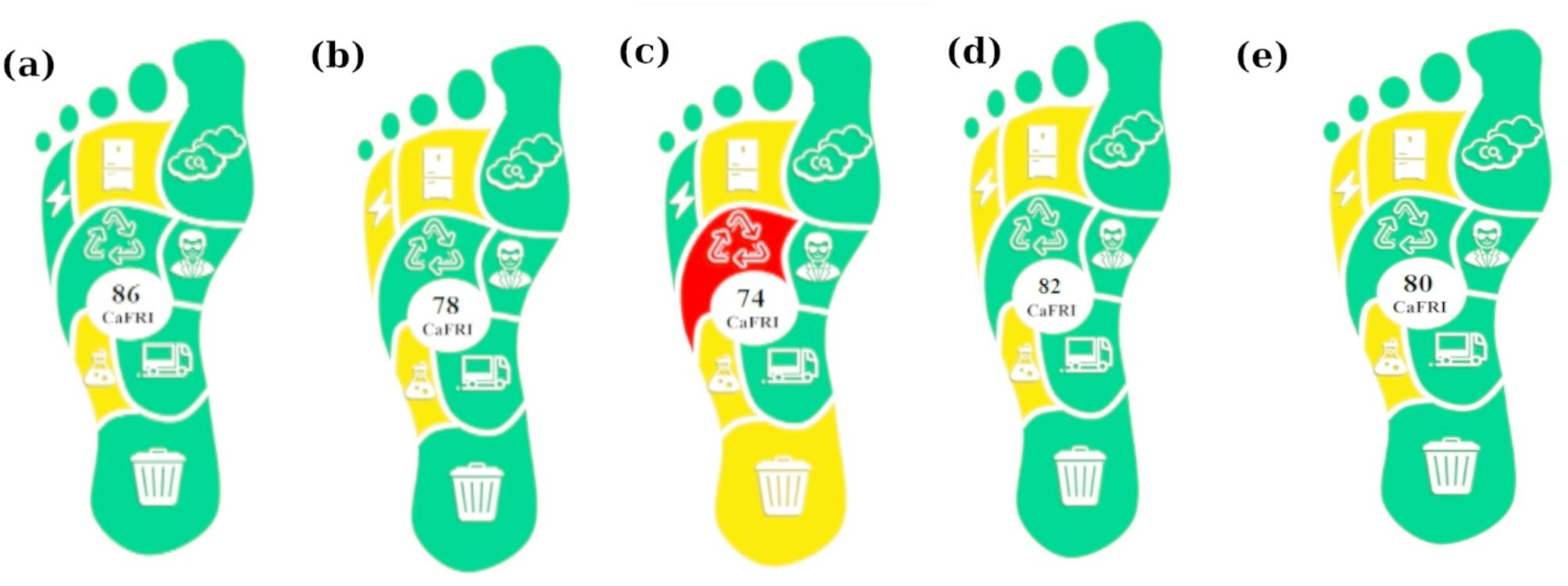
The results of (**a**): the proposed HPLC-DAD method, (**b**): HCT official HPLC method [28], (**c**): CAR official titrimetric method [28], (**d**): Reported HPLC method [35] and (**e**): Reported HPLC method [36] presenting the application of the Carbon Footprint Reduction Index (CaFRI)

The results of the application of CaFRI for the assessment of the proposed HPLC-DAD method as well as the official and reported ones are presented in Fig. 4. The proposed method demonstrates appreciable efforts in reducing the carbon footprint, as indicated by highest cumulative CaFRI score of 86.

### Applicability assessment

The method’s simplicity and practical utility was appraised via two innovative tools; Blue applicability grade index (BAGI) [4], and Click Analytical Chemistry Index (CACI) [61].

BAGI tool is designed to efficiently evaluate the applicability of analytical methods. It serves as a complement to greenness assessment tools, aligning with the “blue” principles of white analytical chemistry [3], which primarily focus on practical considerations. A sequential blue color scale was employed to represent the final score, where distinct shades of dark blue, blue, light blue, and white indicated high, medium, low, and no compliance with the established criteria, respectively. Concerning the total score attained, a value exceeding 60 is recommended for an analytical procedure to be deemed practically applicable. The applicability assessment of the analytical procedure is facilitated via utilizing a corresponding web application (bagi-index.anvil.app).

Whilst, the recently introduced CACI framework offers a practical, efficient, and accessible approach to assess and compare analytical methods, inspired by the simple principles of click chemistry. Significant parameters comprise sample size, preparation, feasibility, applicability, portability, sensitivity, and automation, each scored to highlight the method’s alignment with these principles. Clearly, CACI takes into account various factors that have been neglected in the BAGI metric. The color code of the pictogram in CACI indicates how the method is performing in each aspect. A colored pictogram designates excellent performance, gray indicates intermediate performance, and black represents poor performance or non-compliance with the chosen criteria. The software is available as an open source at bit.ly/CACI2025.

As shown in Fig. S1 and Fig. 3, the suggested method has a higher BAGI (85) as well as CACI (73) scores over the official and reported ones, which confirms to its good applicability.

### In-vitro dissolution monitoring of Co-Dilatrol^®^Tablet

**Fig. 5:**
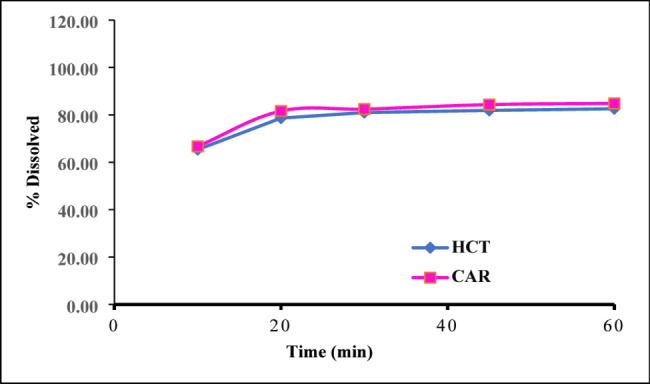
Dissolution profiles of HCT and CAR from Co-Dilatrol^®^ Tablets in 0.1 N HCl as a dissolution medium

Invitro dissolution monitoring is an essential step during drug manufacturing and development to establish the relationship between the in-vitro and in-vivo patterns and to assess the quality of pharmaceutical formulation [39, 62]. Dissolution monitoring of Co-Dilatrol^®^ tablets was performed as recommended by USP [39] utilizing a dissolution medium comprised of 0.1 N HCl. In this research, the release percentage of the cited analytes from Co-Dilatrol^®^Tablets in 0.1 N HCl exceeded 80% after 45 min for HCT and CAR. Hence, the defined acceptance requirements, represented by quantity (Q) of active ingredient dissolved within defined time frame, are satisfied. Dissolution profiles were obtained when plotting % dissolved against time as shown in Fig. 5.

## Conclusion

This pharmaceutical combination exhibits a significant impact, providing dual action as an antihypertensive agent while also attenuating insulin resistance in diabetic individuals. The proposed eco-friendly HPLC-DAD method is efficient, robust, sensitive, and economic. Percent relative standard deviation exhibits lower values, measuring below 2.0%, representing a significant level of precision within the methodology. Short time for analysis (below 9 min), combined with its efficacy, sensitivity, easiness, and operational simplicity, facilitates the application of this methodology for analyzing HCT and CAR in laboratory-prepared mixture with HCT’s related impurities; DSA and CT, in addition to the cited drugs’ analysis in combined pharmaceutical preparation. HPLC methodology provides significant solvent and time savings, consequently diminishing analysis expenses as well as a minimal amount of waste production. Finally, it can be concluded that the suggested eco-friendly HPLC methodology demonstrates superior efficiency and convenience in contrast to established official methods in the impurity profiling of pharmaceutical multi-component mixtures. Hence, the methodology proves effective for conducting the standard quality control investigation of pharmaceuticals within pharmaceutical quality control laboratories and dissolution studies. The assessment of the greenness, blueness and whiteness profile was conducted via up-to-date, user-friendly and reliable metric tools; Furthermore, in-vitro dissolution monitoring of the studied drugs from their formulated drug product was conducted via utilizing the suggested approach. Finally, the studied HPLC-DAD methodology proved its great ability in separating and estimating the four components in short analysis time. So, it has the advantage of the analysis of all these components by one analytical method, which saved time and effort, also gave accurate and reliable results when applied for routine work in pharmaceutical quality control. While the proposed method provides an optimal balance for separating HCT and CT along with CAR and DSA within a reasonable time (less than 9 min) with accepted system suitability parameters, resolution enhancement remains a valuable area for future investigation. This challenging separation, achieved through our proposed approach, continues to inspire researchers to explore innovative solutions that consider not only analytical performance but also applicability, cost-efficiency, and environmental safety, fostering ongoing advancements in this field.

## Electronic supplementary material

Below is the link to the electronic supplementary material.

Supplementary Material 1

## Data Availability

All data generated or analysed during this study is provided within the manuscript or supplementary information files.
