# Supplementary material for: Green HPLC strategy for quantification of carvedilol and hydrochlorothiazide in cardiac medications with in-vitro dissolution kinetics and impurity profiling
Source: BMC Chem. 2025 Jul 3;19(1):187. doi: 10.1186/s13065-025-01559-2 (PMC12224793; doi:10.1186/s13065-025-01559-2)
Supplement: Supplementary file 1 — Supplementary Material 1 [file 13065_2025_1559_MOESM1_ESM.docx]

**(a)**

**(b)**

**(c)**


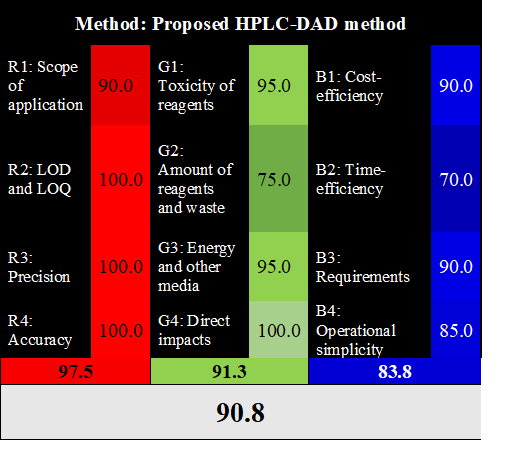

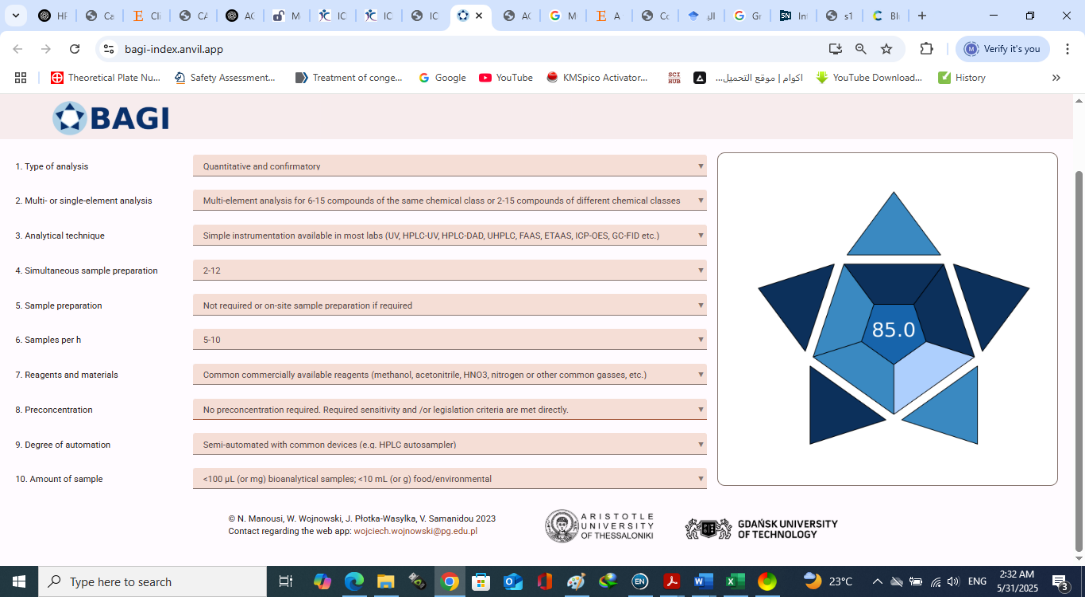

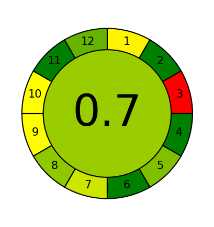

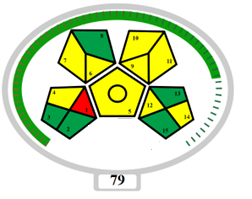

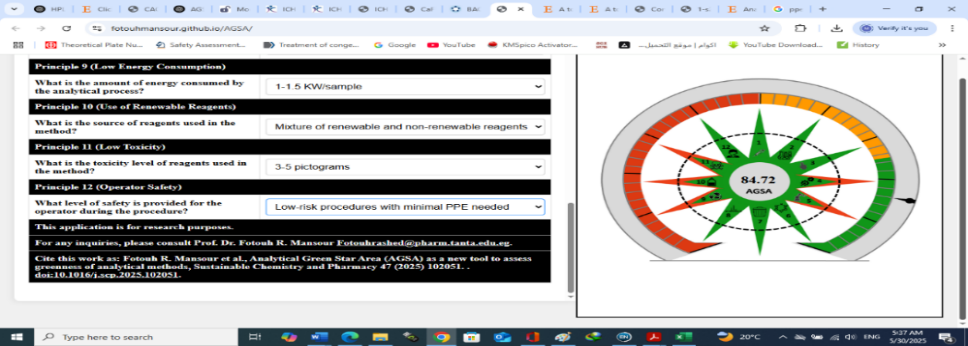

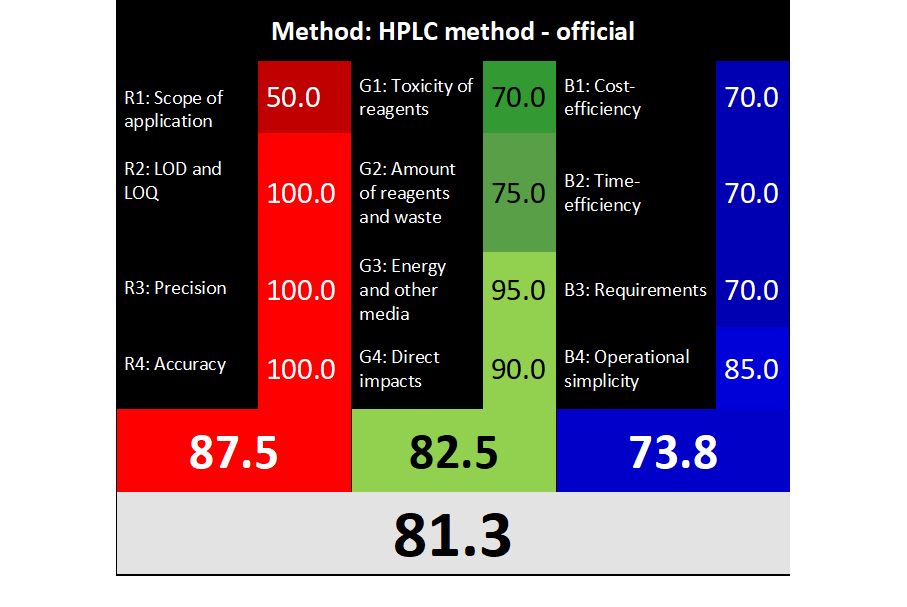

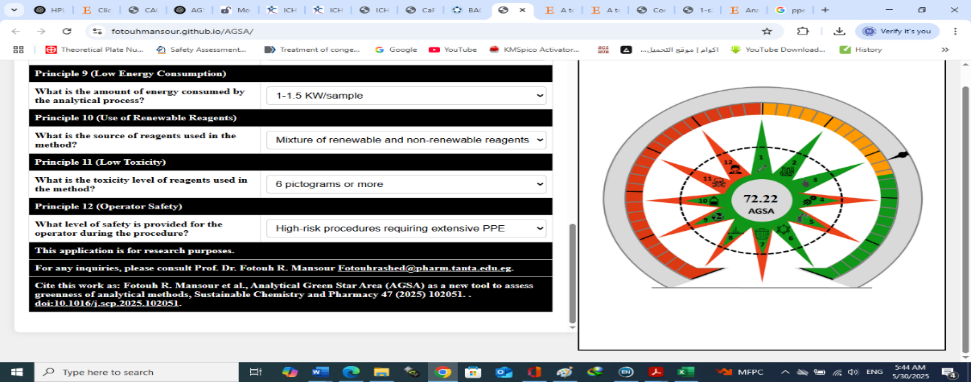

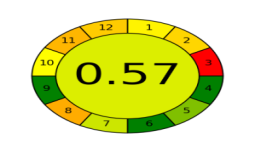

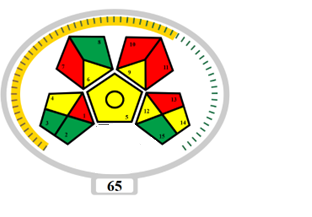

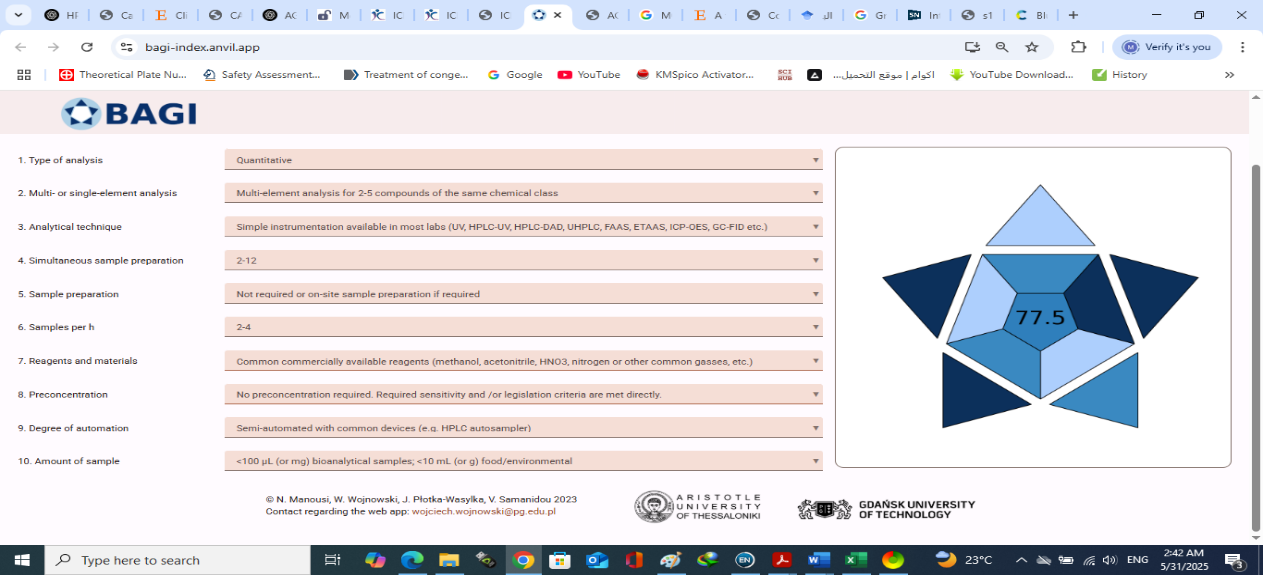

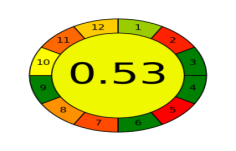

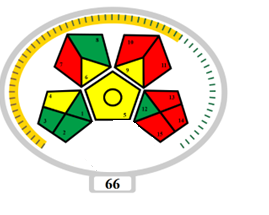

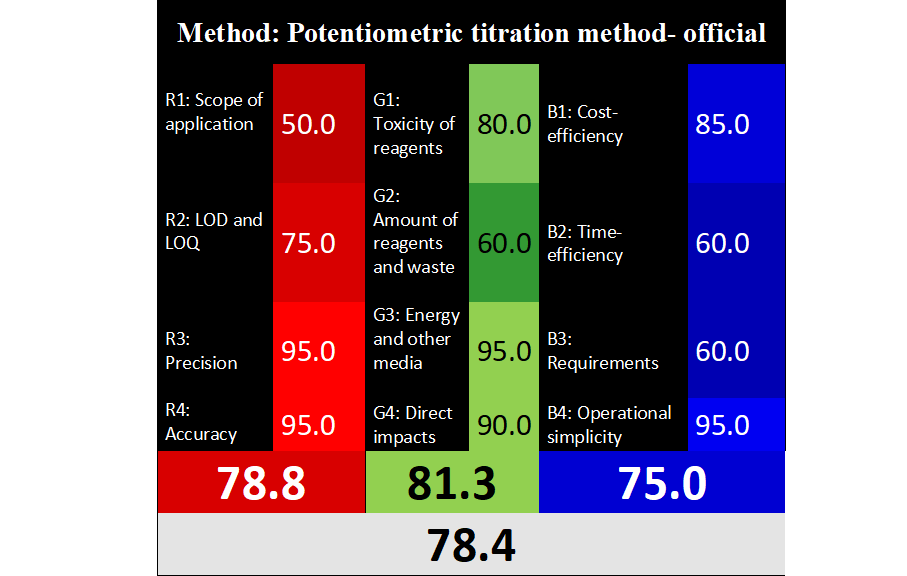

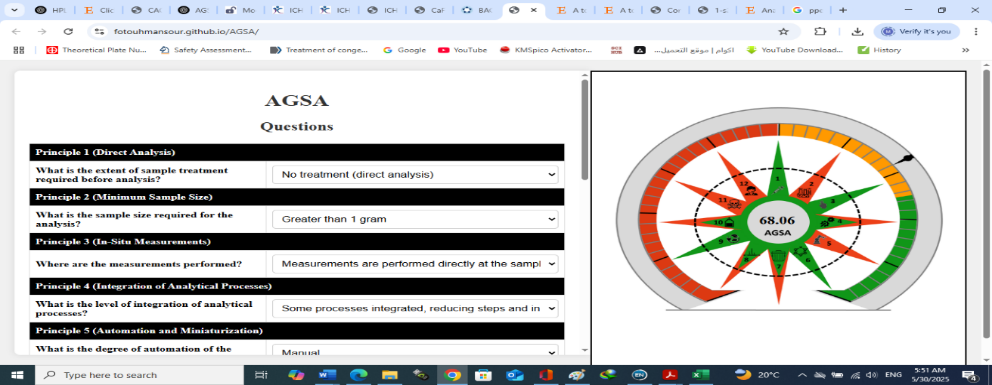

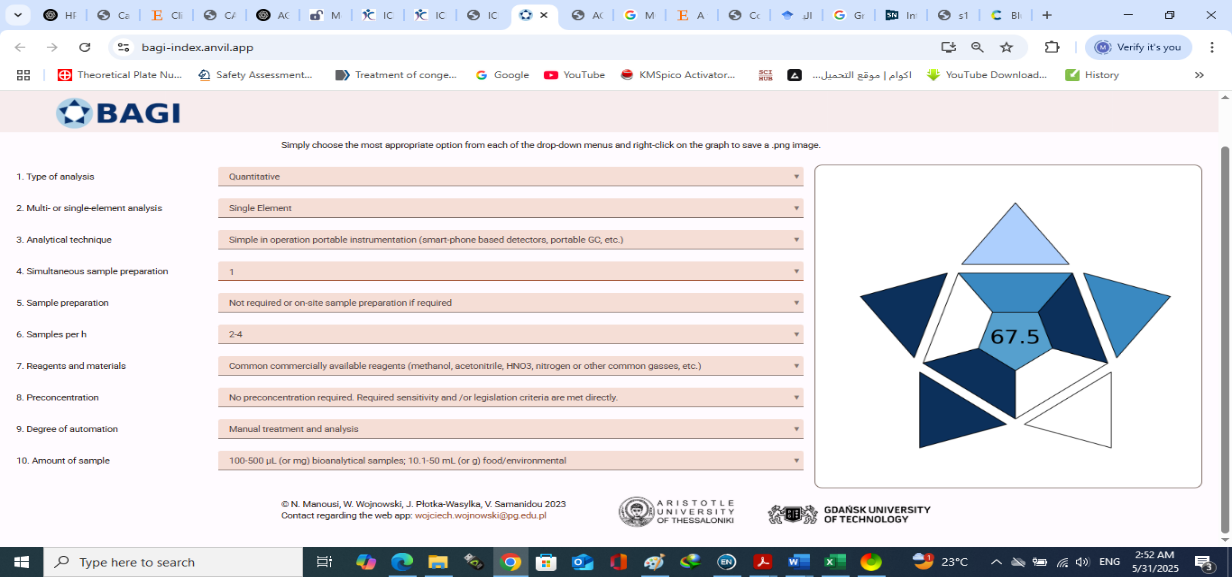

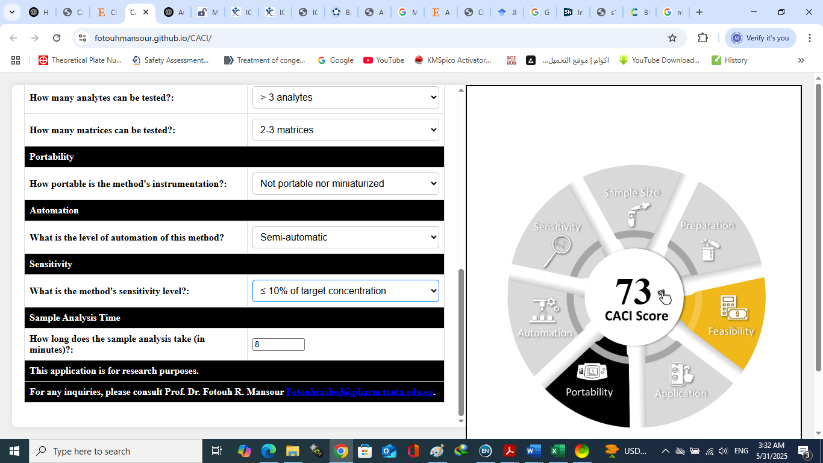

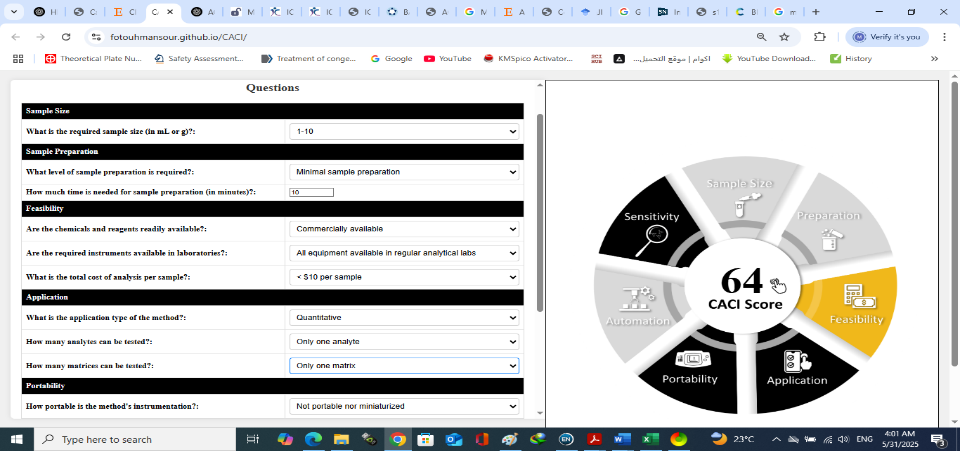

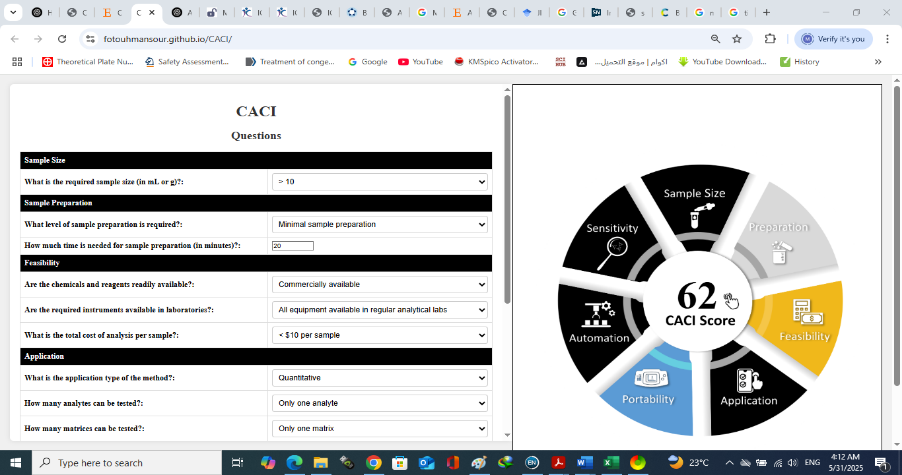


**Fig. S1.** Greenness, blueness and whiteness assessment of (a): the proposed HPLC-DAD method, (b): HCT official HPLC method **[28]** and (c): CAR official titrimetric method **[28],** via AGREE, Mo GAPI, AGSA, BAGI, CACI and RGB 12 tools.

**Table S1:** Determination of HCT and CAR in laboratory prepared mixtures containing different ratios of HCT's related impurities by the proposed HPLC-DAD method.

| HPLC -DAD method | | | | | | | | | | | | | | | | | | | | |
| --- | --- | --- | --- | --- | --- | --- | --- | --- | --- | --- | --- | --- | --- | --- | --- | --- | --- | --- | --- | --- |
|  | **Claimed Conc. (μg/mL)** | | | | | | | | **Found Conc. (μg/mL)** | | | | | | | **Recovery, %^a^** | | | | |
| Impurities (%) | **HCT** | **CAR** | | **CT** | | | **DSA** | | **HCT** | | **CAR** | | **CT** | | **DSA** | **HCT** | **CAR** | | **CT** | **DSA** |
| 0 | 2.50 ^b^ | 5.00 ^b^ | | - | | | - | | 2.51 | | 4.97 | | - | | - | 100.40 | 99.40 | | - | - |
| 10 | 4.50 | 4.50 | | 0.50 | | | 0.50 | | 4.54 | | 4.47 | | 0.49 | | 0.50 | 100.89 | 99.33 | | 98.00 | 100.00 |
| 20 | 4.00 | 4.00 | | 1.00 | | | 1.00 | | 4.03 | | 3.98 | | 1.00 | | 1.01 | 100.75 | 99.50 | | 100.00 | 101.00 |
| 30 | 3.50 | 3.50 | | 1.50 | | | 1.50 | | 3.52 | | 3.48 | | 1.51 | | 1.48 | 100.57 | 99.43 | | 100.67 | 98.67 |
| 40 | 3.00 | 3.00 | | 2.00 | | | 2.00 | | 2.98 | | 3.03 | | 2.01 | | 1.98 | 99.33 | 101.00 | | 100.50 | 99.00 |
| 50 | 2.50 | 2.50 | | 2.50 | | | 2.50 | | 2.52 | | 2.49 | | 2.51 | | 2.48 | 100.80 | 99.60 | | 100.40 | 99.20 |
| 60 | 2.00 | 2.00 | | 3.00 | | | 3.00 | | 2.02 | | 1.99 | | 2.98 | | 2.99 | 101.00 | 99.50 | | 99.33 | 99.67 |
| 70 | 1.50 | 1.50 | | 3.50 | | | 3.50 | | 1.51 | | 1.48 | | 3.52 | | 3.47 | 100.67 | 98.67 | | 100.57 | 99.14 |
| Mean |  | |  | |  |  | |  | |  | |  | |  | | **100.55** | | **99.55** | **99.92** | **99.53** |
| SD |  | |  | |  |  | |  | |  | |  | |  | | **0.53** | | **0.65** | **0.97** | **0.78** |

^a^  Average of three determinations.

^b^ Ratio of HCT and CAR in the pharmaceutical formulation.

**Table S2:** Statistical comparison between the results obtained by the proposed HPLC-DAD method and official BP method [28] for the determination of the proposed mixture in their pure powdered form.

| **Items** | **HCT** | | **CAR** | |
| --- | --- | --- | --- | --- |
|  | **HPLC-DAD method** | **Official method** ^a^ | **HPLC-DAD method** | **Official method** ^b^ |
| **Mean** | 100.02 | 99.88 | 99.58 | 99.93 |
| **SD** | 0.46 | 0.52 | 0.79 | 0.62 |
| **RSD%** | 0.46 | 0.52 | 0.79 | 0.62 |
| **n** | 7 | 4 | 7 | 4 |
| **Variance** | 0.2116 | 0.2704 | 0.6241 | 0.3844 |
| **Student's-t test** | 1.431 (2.262) |  | 1.890 (2.262) |  |
| **F-test** | 1.278 (4.760) |  | 1.624 (8.940) |  |

^a^ For Hydrochlorothiazide: HPLC method using gradient elution of mobile phase A comprising of phosphate buffer, methanol and tetrahydrofuran (94:6:1, by volume) & mobile phase B comprising of phosphate buffer, methanol and tetrahydrofuran (50: 50:5, by volume) (pH 3.2) at flow rate 0.8 mL/min, C_18_ column (4.6 mm × 10 cm) and UV detection at 224 nm.

^b^ For Carvedilol: non-aqueous potentiometric titration method using 0.1 M perchloric acid as a titrant.

**Table S3**: One way ANOVA testing for the proposed and official methods B.P. [28] used for the determination of the cited mixture.

|  | **Source of Variation** | **DF** | **Sum of squares** | **Mean Square** | **F value** | **P- value** |
| --- | --- | --- | --- | --- | --- | --- |
| **HCT** | **Between Groups** | 1 | 0.050 | 0.050 | 0.213 (5.117) | 0.656 |
|  | **Within Groups** | 9 | 2.100 | 0.233 |  |  |
| **CAR** | **Between Groups** | 1 | 0.315 | 0.315 | 0.580 (5.117) | 0.466 |
|  | **Within Groups** | 9 | 4.890 | 0.543 |  |  |

The values between parentheses are the theoretical F values.

The population means are not significantly different.
